# Pharmaceutical Modulation of Intracranial Aneurysm Development and Rupture

**DOI:** 10.3390/jcm13113324

**Published:** 2024-06-05

**Authors:** Alex Crane, Regan M. Shanahan, Joseph S. Hudson, Kamil W. Nowicki, Zachary C. Gersey, Prateek Agarwal, Rachel C. Jacobs, Michael J. Lang, Bradley Gross

**Affiliations:** 1 Department of Neurological Surgery, University of Pittsburgh, Pittsburgh, PA 15213, USAgerseyzc@upmc.edu (Z.C.G.); langmj3@upmc.edu (M.J.L.); 2 Department of Neurosurgery, Yale School of Medicine, New Haven, CT 06510, USA; kamil.nowicki@yale.edu

**Keywords:** aspirin, cerebral aneurysm, intracranial aneurysm, subarachnoid hemorrhage, statin

## Abstract

Management of intracranial aneurysms (IAs) is determined by patient age, risk of rupture, and comorbid conditions. While endovascular and microsurgical interventions offer solutions to mitigate the risk of rupture, pharmacological management strategies may complement these approaches or serve as alternatives in appropriate cases. The pathophysiology of IAs allows for the targeting of inflammation to prevent the development and rupture of IAs. The aim of this review is to provide an updated summary of different pharmaceutical management strategies for IAs. Acetylsalicylic acid and renin-angiotensin-aldosterone system (RAAS) inhibitor antihypertensives have some evidence supporting their protective effect. Studies of selective cyclooxygenase-2 (COX-2) inhibitors, statins, ADP inhibitors, and other metabolism-affecting drugs have demonstrated inconclusive findings regarding their association with aneurysm growth or rupture. In this manuscript, we highlight the evidence supporting each drug’s effectiveness.

## 1. Introduction

Intracranial aneurysms (IAs) are isolated regions of cerebral vessel dilation that carry the risk of rupture and subsequently a potentially lethal subarachnoid hemorrhage (SAH). The most common type of IA is a saccular aneurysm which is distinguished by a “berry-shaped” evagination of the vessel wall [1]. Fusiform aneurysms are dilatations of the entire vessel circumference [1]. Inherent risk factors for IAs include a family history of IAs and female sex, while acquired risk factors include age, hypertension, smoking, and alcohol consumption [2]. An aging population coupled with more frequent use of imaging techniques (CT, MRI) has led to a higher rate of incidental aneurysm diagnoses [3]. IAs are estimated to occur in 3% of the population and are the most common cause of non-traumatic SAH [4,5]. A recent meta-analysis reported a 1.1% rate of rupture in small IAs (≤10 mm) over a mean of 3.7 years of follow-up when treated without endovascular or surgical intervention [6]. In aneurysms where growth is detected, the absolute risk of rupture at two years has been reported to be 6.0% and the mortality of SAH to be 29.0% at five years [7,8]. Treatment of an IA requires thoughtful risk stratification by the managing physician to balance the risk of operative intervention against the risk of conservative treatment to prevent morbidity and mortality in this patient population.

Endovascular and microsurgical techniques carry a risk of morbidity and mortality. In combination with advancing endovascular technology, a pharmacologic option for slowing the progression of aneurysms or preventing recurrent IAs may be a powerful and accessible tool to add to the arsenal of treatment options for patients. Several medications have been investigated for their protective role in IA development and rupture via observational studies; however, the data for most drugs have shown mixed results in clinical settings (Table 1). The purpose of this review is to synthesize recent literature evaluating the effects of pharmacological interventions on the risk of aneurysm development and rupture.

It is important to recognize that pharmaceutical treatment for the prevention of IA progression will likely be most helpful in mitigating the risk of rupture of moderately sized aneurysms. Smaller aneurysms are less likely to rupture and may be surveilled if deemed appropriate by the treating physician. Meanwhile, large aneurysms with a greater risk of rupture may be treated definitively with endovascular or surgical correction, depending on patient presentation [3]. Moderately sized aneurysms are more difficult to assess for surveillance or intervention. If medical therapies were available that could reduce rupture risk, patients with moderately sized aneurysms would benefit greatly. 

## 2. Pharmaceutical Modulation of Intracranial Aneurysms

### 2.1. Pathogenesis of Intracranial Aneurysms

**Figure 1:**
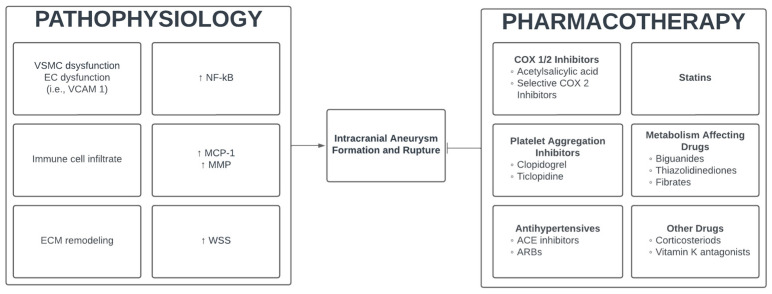
Summary of pathophysiology and pharmacotherapy that could modulate IA formation and rupture.

Aneurysm development depends on a variety of factors including endothelial cell (EC) dysfunction, vessel wall shear stress (WSS), vascular smooth muscle cell (VSMC) dysfunction, extracellular matrix (ECM) remodeling, and immune cell infiltration [9]. WSS has been reported to be higher at branch points in the Circle of Willis where aneurysms frequently develop [10]. Mechanical stress can upregulate the expression of nuclear factor-kβ (NF-kB) which may further induce the expression of cell adhesion molecules, increase chemokines such as monocyte chemoattractant protein-1 (MCP-1), and downregulate important contractile proteins [9]. The resultant endothelial and VSMC dysfunction can lead to apoptosis and immune cell infiltration, creating an ideal inflammatory microenvironment for aneurysm formation. M1 macrophages can further promote degradation of the ECM via matrix metalloproteinase (MMP) production [11]. VSMC death and disruption of the extracellular matrix, including degradation of the elastic lamina, leads to a weakened vessel susceptible to aneurysm formation. Central to aneurysm development is NF-kB, a mediator of inflammation (Figure 1). Several clinical studies have demonstrated greater expression of NF-kB protein in the vessel wall of IAs compared to normal vascular tissue [12,13,14]. Further, many animal models of IA have confirmed the involvement of NF-kB, demonstrating increased mRNA and protein expression [12,15,16]. Thus, in addition to the aforementioned mechanisms of IA pathogenesis, NF-kB is an inflammatory mediator that may be a potential target for drug-based therapy. 

In summary, aneurysms develop when shear stress at branch points in arterial circulation leads to the upregulation of maladaptive genes, such as NF-kB [9]. Consequently, the increased expression of chemokines and cell adhesion molecules leads to the development of an inflammatory local environment. These processes together can cause the degradation of important structural components of the vessel wall, including vascular smooth muscle and extracellular matrix [9]. 

### 2.2. Cyclooxygenase-1 and 2 Inhibitor (NSAIDS)

Attenuation of the NF-kB pathway and reduction of inflammatory mediators is a key goal of IA pharmacotherapy. Acetylsalicylic acid (ASA), commonly referred to as aspirin, has been the most frequently studied pharmacotherapy for the management of IAs. Aspirin has both antipyretic and anti-platelet activity, with its primary clinical benefit stemming from the latter. It inhibits cyclooxygenase-1 (COX1) and COX2, causing the reduced synthesis of prostaglandin H2 and thromboxane A2 [17]. This mitigates the standard platelet aggregation response leading to antithrombotic effects [17]. Additionally, aspirin has been shown to have some benefits outside the realm of its typical antithrombotic utility. Several in vitro and animal models have shown anti-oxidative and anti-inflammatory properties of aspirin, including the inhibition of macrophage populations, tumor necrosis factor-a (TNF-∝), NF-kB expression, and inhibitor of nuclear factor kappa-B kinase subunit beta (IKK-B) [18,19,20]. IKK-B is one of two kinases that facilitate the movement of NF-kB to the nucleus, leading to pro-inflammatory gene activation. ASA-mediated inhibition of IKK-B may prevent such downstream activation of inflammatory pathways [18]. Taken together, these anti-inflammatory properties may reduce matrix degradation, immune cell recruitment, and endothelial dysfunction in aneurysm development. Investigating the precise effects of ASA on IA pathogenesis may be limited by the biochemical properties of eicosanoids, a class of lipid-derived signaling compounds that includes prostaglandins [21]. Precise measurement is limited by short plasma half-lives and the multitude of eicosanoids with similar structures [21,22,23]. However, ASA has broad anti-inflammatory effects that have been demonstrated by measuring other inflammatory markers. Human trials have shown that aspirin may reduce total oxidant status, oxidized low-density lipoprotein (LDL), and C-reactive protein (CRP) [24,25]. LDL accumulation and oxidization may be associated with the degeneration of the aneurysm vessel wall, and CRP is associated with endothelial dysfunction [26,27]. Therefore, a reduction in LDL and CRP secondary to ASA use may have protective effects against aneurysmal development and rupture. 

A quantifiable, protective effect of ASA on IA rupture has yet to be concretely determined, although there are some clinical data supporting the use of ASA in a prophylactic manner. The effects of ASA are best understood as (1) aneurysmal growth prevention and (2) reduced risk of aneurysm rupture. Zanaty et al. analyzed data from patients with multiple small aneurysms (≤5 mm) and found that aspirin had a significant association with a decreased aneurysm growth rate during observation of 146 patients with 229 aneurysms for a minimum of five years follow-up (OR 0.19, CI 0.05–0.63, *p* = 0.007) [28]. In this study, aspirin use was defined as ≥81 mg daily, and adherence to the aspirin regimen was documented in the medical record [28]. A more recent prospective cohort study of 272 patients with unruptured IAs less than 7 mm also found that standard or low dose aspirin greater than three times weekly was associated with a low risk of IA growth (HR 0.29, CI 0.11–0.77, *p* = 0.013) [29]. Importantly, both studies reflect populations with small IAs, and smaller aneurysm size is associated with lower rates of rupture [6].

In 2011, Hasan et al. conducted a nested case-control study with subjects from the International Study of Unruptured Intracranial Aneurysms (ISUIA). The logistic regression results showed that in patients with unruptured IA, aspirin use a minimum of three times weekly had a negative correlation with hemorrhage (aOR = 0.27, 95% CI 0.11–0.67, *p* = 0.03) [30]. Further, a study of 717 patients with cerebral aneurysms reported a much lower frequency of hemorrhagic presentation in patients taking ASA (40% vs. 28%, *p* = 0.016) [31]. More recently, a multicenter study in the UK (n = 2334) with either unruptured IA or aneurysmal SAH found that aspirin use was negatively associated with rupture (OR 0.28, CI 0.20–0.40, *p* < 0.001) [32]. A separate case-control study of 4619 patients with IA reported a significant inverse association of aspirin with IA rupture (OR 0.60, CI 0.45–0.80, *p* < 0.01) [33]. This same study demonstrated a dose-response curve among aspirin users and showed that higher aspirin doses were associated with a decreased risk of IA rupture (unweighted OR 0.72, 95% CI 0.60–0.85; weighted OR 0.65, 95% CI 0.53–0.81; *p* < 0.01) [33]. Lastly, a 2013 study that included 1797 cases of intracerebral hemorrhage (ICH) and 1340 cases of SAH showed no association between ASA and ICH risk (OR 1.06, CI 0.93–1.21) but did show an inverse association between ASA and SAH (OR 0.82, CI 0.67–1.00) [34]. Further regression analysis of 34 of 144 patients with a SAH and a history of aspirin use who were considered long-term users (≥3 years) found a stronger negative correlation with SAH risk (OR 0.63, CI 0.45–0.90), implying that prolonged ASA use could be protective in unruptured IA populations [34].

Further research has also investigated the relationship between aspirin and macrophage infiltration using ferumoxytol-enhanced MRI. Ferumoxytol-enhanced MRIs demonstrate vascular retention properties and can be quantified as a surrogate marker for inflammation [35]. After 3 months of daily aspirin, the signal intensity corresponding to ferumoxytol uptake by macrophages in the vessel wall was decreased from baseline in five patients with IA [35]. This trend was confirmed in later experiments where the signal intensity was stable in a control group of five patients and reduced in six patients treated with aspirin [36]. The authors of this study suggest that the decreased ferumoxytol uptake by macrophages may indicate that aspirin attenuates inflammation in aneurysm walls and therefore may reduce rupture risk [36]. 

Not all studies have supported the beneficial role of aspirin. A 2015 nationwide study in Denmark found that taking aspirin for three or more years was not associated with any increased or decreased risk of SAH (OR 1.13, CI 0.86–1.49), but taking aspirin for less than a month was associated with increased SAH risk (OR 1.75, CI 1.28–2.40) [37]. In a nested case-control study of 2065 patients with SAH selected from the German Pharmacoepidemiological Research Database, aspirin use was associated with an increased risk of SAH (OR 1.5, CI 1.2–2.0, *p* = 0.001) [38]. Lastly, a recent study using subjects from the UK Biobank found no relationship between aspirin use and aneurysmal SAH (HR 1.15, CI 0.91–1.47, *p* = 0.24) but did show that aspirin use was associated with fatal SAH (HR 1.69, CI 1.14–2.51) [39]. The results of these studies raise concerns over the benefit of ASA in patients with IAs and contradict several studies that imply that ASA use is associated with a lower risk of aneurysm growth and rupture. It is important to note that two studies that present negative data are population-level analyses from national databanks. Notably, the cohort study of the UK Biobank defined aspirin use as a binary measure and did not account for dosage or frequency and the nested case-control study which used subjects from a German databank defined aspirin use as current if the patient’s last prescription overlapped with the 7 days preceding hospital admission for SAH [38,39]. Conversely, several of the studies that support aspirin use reflect data from patients whose medication frequency was specified as at least three times weekly. Variability in follow-up criteria, dosing protocols, and patient selection persist as challenges to universal conclusions on ASA prophylaxis in this patient population. 

### 2.3. Selective Cyclooxygenase-2 Inhibitors

Non-steroidal anti-inflammatory drugs (NSAIDs)’ mechanism of action involves the inhibition of the COX1 and COX2 enzymes to mitigate inflammation. Concomitant inhibition of COX1 and COX2 can precipitate unfavorable side effects for patients, including gastric ulcers, hypertension, and acute renal failure [40]. Selective COX2 inhibitors such as celecoxib aim to prevent side effects by avoiding the inhibition of the constitutively expressed COX1 which plays a role in maintaining gastric mucosa [41]. 

The basis of using selective COX2 inhibitors for the treatment and prevention of intracranial aneurysms is supported by animal model data, suggesting these medications attenuate the inflammatory response implicated in IAs. COX-mediated synthesis of prostaglandin E2 causes downstream activation of NF-kB, which has been implicated in aneurysm development [9,42]. Targeting of this pathway is understood to decrease aneurysmal development. Interestingly, animal studies of aortic aneurysms have demonstrated that COX2 inhibition can reduce measures of aneurysm growth and rupture [43,44]. Focusing on intracranial aneurysms, a study by Aoki et al. found that human IA tissues had greater staining of COX2 compared to controls and that in a rat model of IA, there was a significant increase in COX2 mRNA and protein in cerebral artery tissue, 3 months after induction of aneurysm (*p* < 0.01) [45]. Further, inhibition of COX2 prevented aneurysm formation in the same animal model (*p* < 0.05). The authors of this study concluded that COX2 may participate in a positive feedback loop with NF-kB [45]. Moreover, Hasan et al. found that in tissues from patients with IA, COX2 expression was significantly greater in both ruptured and unruptured aneurysms compared to an arterial control (*p* = 0.001) [46]. 

Given the preclinical data specifically implicating COX2 in the pathophysiology of aneurysms it may be reasonable to investigate COX2-specific inhibitors as prophylaxis for aneurysm development. That said, clinical research in this area has been limited, with the focus on aspirin, a nonspecific inhibitor of COX enzymes that has shown significant promise. A recent study of 1419 patients with saccular IAs found that neither treatment with COX2 selective inhibitors (HR 0.63, CI 0.29–1.39, *p* = 0.249) nor NSAIDS (HR 1.11, CI 0.50–2.45, *p* = 0.805) reduced de novo IA formation [47]. Risselada et al. found that the use of COX2 selective inhibitors was positively correlated with SAH (OR 2.35, CI 1.27–4.36) [48]. Additionally, a meta-analysis found that non-aspirin NSAIDS (OR 1.73, CI 1.07–2.79) and selective COX2 inhibitors (OR 2.04, CI 1.24–3.35) were positively associated with aneurysmal SAH [49]. There is an apparent disconnect between pre-clinical and human trials, and it is important to note that the efficacy of COX2 inhibition is limited by its inhibition of prostacyclin which has beneficial vasodilatory effects and limits vascular remodeling [50]. The current body of research is limited, and prospective investigations can delineate the protective effect of COX2 inhibition. 

### 2.4. Platelet Aggregation Inhibitors

Clopidogrel, a thienopyridine, acts by selectively inhibiting adenosine diphosphate-mediated platelet aggregation [51]. More specifically, these medications are antagonists of the P2Y12 platelet receptor and harbor some anti-inflammatory properties. In a rat model of renal inflammation, treatment with clopidogrel reduced the expression of many pro-inflammatory factors including MCP-1 (*p* < 0.05) [52]. MCP-1 may be a pharmacologic target in the modulation of IA formation as animal models have reaffirmed the role of MCP-1 in cerebral IA development [53]. 

A recent retrospective study of 921 patients with IAs found that exposure to clopidogrel reduced the likelihood of rupture (6.6% vs. 23.5%, *p* = 0.001) [54]. A case-control study of 1004 SAH cases found a positive correlation between platelet aggregation inhibitors and risk of SAH (OR 1.32, CI 1.02–1.70) [48]. However, this association was not significant in a further case-crossover analysis and the study did not differentiate between classes of platelet-aggregation inhibitors, thus clopidogrel, ticlopidine, aspirin, dipyridamole, and carbasalate calcium were all included together [48]. To add to the conflicting nature of current data, a nationwide case-control study from Denmark (n = 5834) reported data indicating that clopidogrel was only associated with an increased risk of SAH in the short term [37]. There was a positive association between clopidogrel use and risk of SAH in patients taking the drug for less than a month (OR 2.33, CI 1.02–5.35) and between 2 and 3 months (OR 2.40, CI 1.20–4.81); however, this association was not significant beyond 3 months of use [37].

There is no clear answer on whether ADP receptor inhibitors have a protective or harmful effect on IA aneurysm rupture. The current data available are a small mix of observational studies demonstrating conflicting data. Higher strength evidence is needed in the form of high-powered case-control studies and if possible, randomized controlled trials, to delineate what role, if any, ADP receptor inhibitors can play in aneurysm management. 

### 2.5. Antihypertensives

Hypertension is one of the most prominent risk factors for IA development and rupture [55,56,57]. Additionally, hemodynamic stress and renin–angiotensin–aldosterone system (RAAS) activation-induced inflammatory responses can perpetrate IA pathophysiology [55]. Chronic hypertension is accompanied by a variety of histopathological changes including altered intimal layer thickness, tunica media necrosis, and degeneration of the internal elastic lamina, which all compromise the structural integrity of the cerebral vasculature [58]. Hypertensive patients have a 2.6 times greater risk of ruptured IA when compared to non-hypertensive patients per a cross-sectional study of 29 patients [59]. A larger retrospective study of 3965 patients also found hypertension to be significantly associated with increased IA rupture risk (OR 2.55, CI 2.16–3.03) [60]. Anti-hypertensive medications have emerged as successful pharmacotherapies to reduce rupture risk in IA patients. A mouse model of intracranial aneurysms demonstrated that controlling blood pressure with hydralazine significantly reduced IA rupture rate (*p* < 0.05) [55]. Moreover, this study showed a dose-dependent relationship between hydralazine and IA rupture rate. Furthermore, initiation of captopril (angiotensin-converting enzyme inhibitor) reduced the incidence of rupture (*p* < 0.05) and improved survival (*p* < 0.05) in mice with IAs. Lastly, this study also investigated the roles of the local RAAS on vascular wall integrity. The results demonstrated that angiotensin-converting enzyme (ACE) inhibitors and angiotensin II type 1 receptor antagonists (ARB) reduced IA rupture rate without altering systemic hypertension, which further suggests the activation of a regional vascular RAAS system in IA rupture [55].

In a recent multi-center analysis (n = 3044), RAAS inhibitors were significantly associated with reduced rupture risk versus non-RAAS inhibitors (OR: 0.490, 95% CI: 0.402–0.597, *p* = 0.000) [61]. Individually, treatment with ACE inhibitors (OR: 0.559, 95% CI: 0.442–0.709, *p* = 0.000) or ARBs (OR: 0.414, 95% CI: 0.315–0.542, *p* = 0.000) suggested a lower risk of rupture. Notably, the association between RAAS inhibitors and reduced rupture risk was independent of blood pressure control, suggesting that RAAS inhibitors are the optimal blood pressure medications to prevent IA rupture in hypertensive patient populations [61].

### 2.6. Hydroxymethylglutaryl-CoA Reductase Inhibitors (Statins)

Hydroxymethylglutaryl-CoA (HMG-CoA) reductase inhibitors are a class of medications commonly referred to as statins. In addition to lowering LDL-cholesterol via synthesis inhibition, it has been proposed that statins may improve endothelial function through anti-inflammatory properties [62]. On a pathophysiological level, a trial of 17,802 healthy participants with elevated CRP levels treated with 20 mg of rosuvastatin daily showed significantly reduced levels of CRP (37% reduction, *p* < 0.001) [63]. CRP is associated with reduced endothelial vasoreactivity, and pharmacologic lowering of this marker could possibly reduce endothelial dysfunction, a process implicated in aneurysm development and rupture [27,63]. Landmesser et al. further demonstrated that compared to ezetimibe (selective cholesterol-absorption inhibitor), simvastatin had superior effects on arterial dilation. Here, improved arterial dilation demonstrated that statins disrupt aberrant endothelial function and that the mechanism of action in cholesterol-lowering drugs dictates the impact on vascular response [64]. An in vitro model demonstrated that incubation of monocytes in cerivastatin reduced adhesion to vascular endothelium [65]. Lastly, a clinical trial of 146 patients found that MCP-1 was reduced by 28% from baseline in patients treated with 1 mg/day pitavastatin and reduced by 11% in patients treated with 5 mg/day atorvastatin [66]. 

There are a few studies that provide data supporting statin use in patients with IAs. A multicenter study from Japan selected 117 cases of ruptured IAs and found an inverse relationship between statin use and aneurysmal rupture (aOR 0.30, CI 0.14–0.66) [67]. Another study of 4701 patients with IAs reported that the use of lipid-lowering medications was found to be inversely correlated with rupture (OR 0.58, CI 0.47–0.71, *p* < 0.01) [68]. Importantly, of the 4701 patients, 1129 were using lipid-lowering agents, and 937 of those patients were using statins only [68]. To add to the evidence backing statin use in IA patients, a cross-sectional study of 310 patients with ruptured IA and 887 patients with unruptured IA reported an inverse association of statin usage with rupture (OR 0.54, CI 0.38–0.77, *p* = 0.0008) [69]. 

Further research has sought to examine the effects of statin use on the pathogenesis of aneurysm development in patients with IA. Aneurysm wall enhancement (WE), measured via MRI, has emerged as one such technique to quantify the effect of treatments on aneurysmal stability. Aneurysmal WE is indicative of wall inflammation and confers an increased risk of rupture [70]. A recent study assessed 127 fusiform aneurysms and found that statin use was significantly correlated with a decreased WE (β = −0.236, *p* = 0.007) [71]. Moreover, a randomized controlled trial of 60 patients with unruptured IAs larger than 3 mm found that 20 mg of atorvastatin daily led to a decrease in WE as compared to baseline (*p* = 0.039) and a placebo group (*p* = 0.006), after 6 months of treatment [72]. Interestingly, there was no difference in aneurysm size in atorvastatin or placebo-treated groups [72]. 

Despite these studies which support the clinical use of statins in patients with IAs, several data suggest otherwise. One study examined the combined effect of statin and aspirin treatment on 194 ruptured IAs and 214 unruptured IAs and found that combined medical therapy was correlated with unruptured status (aOR 5.01, CI 1.37–18.33, *p* = 0.015), but statins alone were not found to have any association with IA rupture (aOR:1.65, CI 0.83–3.31, *p* = 0.155) [73]. A randomized controlled trial (n = 209) investigating the impact of 10 mg of atorvastatin daily, found no significant difference in aneurysm growth, rupture, or “new bleb formation” over a mean imaging follow-up period of 32.4 months (Log-rank *p* = 0.359) [74]. Of note, the systolic pressure, a known risk factor for aneurysm rupture, was significantly higher in the statin-treated group (*p* = 0.03) [74,75]. Further analysis is needed to delineate the impact of statins on aneurysmal growth while controlling for confounding comorbidities. A case-control study of 1200 patients with confirmed IA found no correlation between statin use and IA development (OR 1.08, CI 0.69–1.69) [76]. A separate study of patients with unruptured IAs (n = 28,931) reported that statin use was not associated with the risk of SAH (OR 1.03, CI 0.86–1.23, *p* = 0.730) [77]. Lastly, a recent study compared a patient cohort with IAs (n = 1960) to matched controls and found that statins were associated with a higher risk of IA development (aOR 1.34, CI 1.02–1.78) [78]. These results should be interpreted cautiously though, considering the likelihood of patients who are prescribed statins having other risk factors for aneurysm development (i.e., hypertension). Conversely, in the same study, a multivariate analysis of the IA patient cohort alone found that patients with ruptured IAs were less likely to be treated with statins (aOR 0.62, CI 0.47–0.81) [78]. Conflicting conclusions surrounding the use of statin use for IA management are complicated by the burden of comorbid conditions and medication compliance. A refined understanding of the role of statins in aneurysmal rupture prevention will require further clinical investigation with judicious controls on comorbid conditions. 

### 2.7. Metabolism Affecting Drugs

Biguanides are a class of medications used to treat diabetes mellitus. Included in this class of drugs is metformin, the most prescribed anti-diabetic medication worldwide [79]. The precise mechanism of biguanides is yet unclear; however, it is evident that these drugs inhibit hepatic gluconeogenesis [80]. Further, given the pleiotropic effects of metformin, many groups have sought to investigate its potential benefit in different disease models. In a rat model of elastase-induced IA, it was found that metformin decreased the rate of IA rupture (*p* < 0.05) [81]. Furthermore, a meta-analysis examined the data from two studies assessing the correlation between biguanides and aneurysmal SAH, and found an inverse association with SAH (OR 0.57, CI 0.34–0.96) [49].

Thiazolidinediones (TZDs) are medications also used to treat type 2 diabetes mellitus that reverse insulin resistance and reduce the progression of metabolic syndrome. TZDs decrease hepatic gluconeogenesis, lower insulin levels, and decrease triglycerides [82]. On a cellular level, TZDs are peroxisome proliferator-activated regulator-y (PPAR-y) agonists [82]. It is understood that PPAR-y is expressed by both macrophages and VSMCs which are implicated in the pathophysiology of IA development [82]. Current research in animal models suggests that dysregulated PPAR-y may be implicated in pro-inflammatory states and ultimately, aneurysm development. In a mouse model of intracranial aneurysm, a PPAR-y antagonist increased the incidence of subarachnoid hemorrhage (*p* < 0.05) [83]. The same study also showed that transgenic mice with a dominant-negative smooth muscle-specific PPAR-y mutation, had significantly greater rates of aneurysm formation (*p* < 0.05) and rupture (*p* = 0.05) [83]. From a pharmacotherapy perspective, pioglitazone reduced the incidence of ruptured aneurysms compared to vehicle controls (*p* < 0.05) [84]. Further, in a mouse model of abdominal aortic aneurysm, rosiglitazone inhibited fatal aneurysm rupture (*p* = 0.0013) and reduced the maximal aortic dilatation (*p* < 0.0001) [85].

Fibrates are medications used to reduce serum triglyceride levels. They induce lipolysis, hepatic fatty acid uptake and increase reverse cholesterol transport [86]. Fibrates are also activators of peroxisome proliferator-activated receptor-a (PPAR-a) [87]. While there is limited literature on the effects of fibrates in cerebral aneurysms, a few basic science and clinical studies have investigated their use for aortic aneurysms and suggested a beneficial effect [87,88]. Two randomized controlled trials examining fibrate use in abdominal aneurysm patients have also been conducted [89,90]. Further investigation is required to characterize the effect of fibrates on IA development and rupture risk.

### 2.8. Other Medications 

Synthetic glucocorticoids have potent anti-inflammatory effects that are mediated through the binding of the glucocorticoid receptor (GR) [91]. The ligand–receptor complex translocates to the nucleus and subsequently suppresses or elicits transcription of various genes. A few pro-inflammatory factors that are negatively regulated include factors implicated in IA pathogenesis, such as COX2, inducible nitric oxide synthase (iNOS), and TNF-a [91]. The role of corticosteroids in the pharmacological modulation of IA is underreported but they may modulate the inflammatory factors contributing to IA development and rupture. An observational study of 1158 patients presenting with SAH and confirmed IA found that SAH was significantly associated with the composite outcome of a history of corticosteroid use or a medical condition that may require the use of corticosteroids (OR 1.67, CI 1.09–2.54, *p* = 0.0l6) [92]. Additionally, autoimmune conditions modulate pro-inflammatory states and are also associated with smaller ruptured aneurysm size (*p* = 0.03) [93].

**Table 1:** Summary of clinical studies examining pharmaceutical modulation of intracranial aneurysms.

Study Author (Year)	No. of Pts	Medication	Association
Zanaty et al. (2020) [28]	N = 146	ASA	Decreased IA growth (OR 0.19, CI 0.05–0.63, *p* = 0.007)
Weng et al. (2020) [29]	N = 272	ASA	Decreased IA growth (HR 0.29, CI 0.11–0.77, *p* = 0.013)
Hasan et al. (2011) [30]	N = 271	ASA	Decreased odds of hemorrhage (aOR 0.27, CI 0.11–0.67, *p* = 0.03)
Gross et al. (2014) [31]	N = 717	ASA	Decreased rate of hemorrhage (40% vs. 28%, *p* = 0.016)
Hostettler et al. (2018) [32]	N = 2334	ASA	Negative association with rupture (OR 0.28, CI 0.20–0.40, *p* < 0.001)
Can et al. (2018) [33]	N = 4619	ASA	Negative association with rupture (OR 0.60, CI 0.45–0.80, *p* < 0.01) Dose response (OR 0.65, CI 0.53–0.81, *p* < 0.01)
Garcia-Rodriguez et al. (2013) [34]	N = 1340	ASA > 3 years	Decreased risk of SAH (OR 0.63, CI 0.45–0.90)
Garbe et al. (2013) [38]	N = 2065	ASA	Increased risk of SAH (OR 1.5, CI 1.2–2.0, *p* = 0.001)
Ewbank et al. (2023) [39]	N = 541	ASA	No association with SAH (HR 1.15, CI 0.91–1.47, *p* = 0.24)
Pottegård et al. (2015) [37]	N = 5834	ASA < 1 month	Increased risk of SAH (OR 1.75, CI 1.28–2.40)
		ASA > 3 years	No association with SAH (OR 1.13, CI 0.86–1.49)
Raisanen et al. (2022) [47]	N = 1419	COX2i	No association with IA formation (HR 0.63, CI 0.29–1.39, *p* = 0.249)
Risselada et al. (2011) [48]	N = 1004	COX2i	Positive association with SAH (OR 2.35, CI 1.27–4.36)
Pottegård et al. (2015) [37]	N = 5834	Clopidogrel < 1 month	Positive association with SAH (OR 2.33, CI 1.02–5.35), no significant relationship in long-term users.
Hudson et al. (2023) [54]	N = 921	Clopidogrel	Lower likelihood of rupture (6.6% vs. 23.5%, *p* = 0.001)
Risselada et al. (2011) [48]	N = 1004	Platelet Aggregation Inhibitors	Positive association with SAH (OR 1.32, CI 1.02–1.70) in case-control study, significance lost in case-crossover analysis.
Zhong et al. (2022) [61]	N = 3044	ACEi	Negative association with rupture (OR 0.559, CI 0.442–0.709, *p* = 0.000)
		ARBs	Negative association with rupture (OR 0.414, CI 0.315–0.542, *p* = 0.000)
Yoshimura et al. (2014) [67]	N = 421	Statins	Negative association with rupture (aOR 0.30, CI 0.14–0.66)
Shimizu et al. (2021) [69]	N = 1197	Statins	Negative association with rupture (OR 0.54, CI 0.38–0.77, *p* = 0.0008)
Terceno et al. (2021) [73]	N = 368	Statins	No association with rupture (aOR 1.65, CI 0.83–3.31, *p* = 0.155)
Yoshida et al. (2021) [74]	N = 209	Statins	No difference in IA growth, rupture, or “new bleb formation” (Log-rank *p* = 0.359)
Marbacher et al. (2012) [76]	N = 300	Statins	No association with IA formation (OR 1.08, CI 0.69–1.69, *p* = 0.74)
Bekelis et al. (2015) [77]	N = 28,931	Statins	No association with SAH (OR 1.03, CI 0.86–1.23, *p* = 0.730)
Jabbarli et al. (2023) [78]	N = 1960	Statins	Positive association with IA formation (aOR 1.34, CI 1.02–1.78)
	N = 2446	Statins	Negative association with rupture (aOR 0.62, CI 0.47–0.81)
Can et al. (2018) [68]	N = 4701	Lipid-Lowering Medications	Negative association with rupture (OR 0.58, CI 0.47–0.71, *p* < 0.01)
Ruigrok et al. (2006) [92]	N = 1158	Corticosteroids	Composite outcome of corticosteroids or a medical condition that may be treated with corticosteroids had a positive association with SAH (OR 1.67, CI 1.09–2.54, *p* = 0.016)
Pottegård et al. (2015) [37]	N = 5834	Vitamin-K Antagonists	No association with SAH (OR 1.24, CI 0.86–1.77) in long-term users (>3 years)
Garbe et al. (2013) [38]	N = 2065	Vitamin-K Antagonists	Positive association with SAH (OR 1.7, CI 1.3–2.3, *p* < 0.001)
Risselada et al. (2011) [48]	N = 1004	Vitamin-K Antagonists	Positive association with SAH (OR 2.90, CI 1.27–6.65) in case-crossover, not significant in case-control

Lastly, vitamin K antagonists are anti-coagulant medications that act by inhibiting the vitamin K epoxide reductase complex [94]. The prevention of the gamma-carboxylation of vitamin-K-dependent coagulation factors reduces thrombosis risk [94]. A 2011 study of 1004 SAH cases found no association between the risk of SAH and vitamin K antagonist use (OR 1.29, CI 0.89–1.87) in a case-control analysis but did find a significant positive association in a case-crossover analysis (OR 2.90, CI 1.27–6.65) [48]. Conversely, a later case-control study of 2065 cases of SAH found that the risk of SAH was positively associated with phenprocoumon, a vitamin K antagonist (OR 1.7, CI 1.3–2.3, *p* < 0.001) [38]. Lastly, Pottegard et al. found that vitamin K antagonists were not significantly correlated with SAH with long-term (>3 years) use (aOR 1.24, CI 0.86–1.77) [37]. 

Recent research on the forefront of aneurysm pathophysiology has demonstrated a possible role for the tyrosine kinase inhibitor, sunitinib [95]. The authors produced a mouse model of basilar artery aneurysm by overexpressing a mutated platelet-derived growth factor B, and further showed that treatment with sunitinib significantly reduced arterial cross-sectional area (*p* < 0.05) [95]. Similarly, in a rat model of intracranial aneurysm, erlotinib was shown to reduce the rate of aneurysm development [96].

The utility of corticosteroids and vitamin-K antagonists in the prevention of IA development and rupture is unclear and future studies that adequately control for underlying disease states are best suited to characterize their impact on IA development and rupture. Pharmaceuticals tested in the pre-clinical space such as tyrosine kinase inhibitors may lead to new discoveries in the pathogenesis of IAs which could further the use of prophylactic medications for IA progression.

## 3. Conclusions

While there is an abundance of literature examining the effects of acetylsalicylic acid exposure on aneurysm growth and rupture, the data are inconsistent. Robust clinical studies of populations with medium-sized aneurysms may reveal a more niche role for ASA in the treatment of IAs. Further research is needed to fully elucidate the effects of statins, COX2 inhibitors, and ADP inhibitors, among other metabolism-affecting drugs, on the development, growth, and rupture of intracranial aneurysms.
